# Supplementary material for: Association between meteorological factors and the prevalence dynamics of Japanese encephalitis
Source: PLoS One. 2021 Mar 3;16(3):e0247980. doi: 10.1371/journal.pone.0247980 (PMC7928514; doi:10.1371/journal.pone.0247980)
Supplement: S1 Table — (DOCX) [file pone.0247980.s001.docx]

**S1 Table. Descriptive analysis of variables from 2007 to 2019 in Chongqing.**

|  | Mean | Minimum | P_5_ | P_50_ | P_75_ | Maximum |
| --- | --- | --- | --- | --- | --- | --- |
| *D* | 9.89 | 0.00 | 0.00 | 0.00 | 1.5 | 198.00 |
| *T*_mean_(°C) | 17.99 | 3.87 | 6.95 | 18.52 | 24.00 | 30.40 |
| *T*_max_(°C) | 22.25 | 6.26 | 9.88 | 22.78 | 28.60 | 35.80 |
| *T*_min_ (°C) | 14.96 | 2.23 | 4.81 | 15.38 | 21.13 | 25.90 |
| *H*_mean_ (%) | 77.84 | 61.20 | 69.07 | 78.48 | 81.63 | 87.23 |
| *P* (mm) | 1125.89 | 78.50 | 147.40 | 1053.30 | 1611.05 | 4211.90 |
| CDH | 0.41 | 0.00 | 0.00 | 0.00 | 0.15 | 16.30 |
| CDL | 2.07 | 0.00 | 0.00 | 0.00 | 0.75 | 49.10 |
| DMSH | 20.09 | 0.00 | 0.00 | 0.00 | 34.30 | 139.80 |
| DMSL | 75.23 | 0.00 | 0.00 | 0.00 | 140.70 | 441.80 |

CDH: The *C. tritaeniorhynchus* density in human houses

CDL: The *C. tritaeniorhynchus* density in livestock sheds

DMSH: The density of all mosquito species in human houses

DMSL: The density of all mosquito species in livestock sheds
