# Supplementary material for: Association between meteorological factors and the prevalence dynamics of Japanese encephalitis
Source: PLoS One. 2021 Mar 3;16(3):e0247980. doi: 10.1371/journal.pone.0247980 (PMC7928514; doi:10.1371/journal.pone.0247980)
Supplement: S2 Table — (DOCX) [file pone.0247980.s002.docx]

**S2 Table. Correlation analysis using Pearson's correlation test.**

|  | *D* | *T*_mean_ | *T*_max_ | *T*_min_ | *H*_mean_ | *P* | CDH | CDL | DMSH | DMSL |
| --- | --- | --- | --- | --- | --- | --- | --- | --- | --- | --- |
| *D* | 1* |  |  |  |  |  |  |  |  |  |
| *T*_mean_ | 0.44* | 1* |  |  |  |  |  |  |  |  |
| T_max_ | 0.44* | 0.99* | 1* |  |  |  |  |  |  |  |
| T_min_ | 0.45* | 0.99* | 0.98* | 1* |  |  |  |  |  |  |
| *H*_mean_ | -0.22* | -0.35* | -0.40* | -0.28* | 1* |  |  |  |  |  |
| *P* | 0.29* | 0.76* | 0.74* | 0.77* | 0.01 | 1* |  |  |  |  |
| CDH | 0.18* | 0.31* | 0.32* | 0.31* | -0.27* | 0.12 | 1* |  |  |  |
| CDL | 0.21* | 0.38* | 0.38* | 0.38* | -0.29* | 0.18* | 0.87* | 1* |  |  |
| DMSH | 0.44* | 0.70* | 0.71* | 0.71* | -0.16 | 0.59* | 0.39* | 0.37* | 1* |  |
| DMSL | 0.41* | 0.78* | 0.77* | 0.79* | -0.31* | 0.59* | 0.56* | 0.68* | 0.72* | 1* |

CDH: The *C. tritaeniorhynchus* density in human houses

CDL: The *C. tritaeniorhynchus* density in livestock sheds

DMSH: The density of all mosquito species in human houses

DMSL: The density of all mosquito species in livestock sheds
