# Supplementary material for: Association between meteorological factors and the prevalence dynamics of Japanese encephalitis
Source: PLoS One. 2021 Mar 3;16(3):e0247980. doi: 10.1371/journal.pone.0247980 (PMC7928514; doi:10.1371/journal.pone.0247980)
Supplement: S3 Table — (DOCX) [file pone.0247980.s003.docx]

**S3 Table. Comparison of model performance using the Root Mean Square Error (RMSE).**

| Model | Training set (2008-2018) | Testing set (2019) |
| --- | --- | --- |
| Long Short-Term Memory  (LSTM) | **18.87** | **3.23** |
| Back Propagation Neural Networks  (BPNN) | 58.69 | 18.09 |
| Gradient Boosting Machine  (GBM) | 22.55 | 12.39 |
| Support Vactor Regression  (SVR) | 58.52 | 16.19 |
| Generalized Additive Model  (GAM) | 58.88 | 30.29 |
