# Supplementary material for: Association between meteorological factors and the prevalence dynamics of Japanese encephalitis
Source: PLoS One. 2021 Mar 3;16(3):e0247980. doi: 10.1371/journal.pone.0247980 (PMC7928514; doi:10.1371/journal.pone.0247980)
Supplement: Attachment — Submitted filename: Response to Reviewers.docx [file pone.0247980.s004.docx]

November 3, 2020

*PLoS ONE*

Dear Editors,

On behalf of my co-authors, we thank you very much for giving us an opportunity to revise our manuscript, we appreciate the editor for their meticulous working attitude.

We redrew Figure 1 using the ArcGIS (version 10.6). Therefore, the figure has no copyright issues.

I look forward to hearing from you again.

Best regards,

**Taotian Tu** (taotiantu@sina.cn)

Chongqing Municipal Center for Disease Control and Prevention, Chongqing, China;

**Wenge Tang** (twg@cqcdc.org)

Chongqing Municipal Center for Disease Control and Prevention, Chongqing, China.

Response to Reviewer #1:

We would like to thank the reviewer for careful and thorough reading of this manuscript and for the thoughtful comments and constructive suggestions, which help to improve the quality of this manuscript.

At the following, the points mentioned by the reviewers will be discussed:

1. The term climate factor should be replaced with ‘meteorological factor’.

[Reply] Thank you for your advice. After careful consideration, we also think it is more reasonable to change " climate factor " to " meteorological factor ". We have made changes in main text (line1, 23, 26, 82, 237, 301).

2. The author applies GAM to study the relationship for vector density, and long short term memory model (LSTM) for JE incidence with met. Parameters. No justification for adopting two different modeling aspect.

[Reply] Thank you for your advice. This study analyzed the correlation between meteorological variables and the incidence of JE by using the ZIGAM. With the help of the research results, we use LSTM to predict the occurrence of JE in the next year, so that the prediction will be more accurate. We added a comparison of the LSTM and GAM models in S3 table and Figure 5.

3. The predictive ability of the model should be written abstract line, and author avoid to do this.

[Reply] Thank you for your advice. We have made changes in the figure of the predictive ability of the model (Figure 5).

4. The Introduction section is not structured. Globally published literature is missing. The hierarchy should be, global aspect, then continental, then country, then province/district. The author fails to perform this hierarchy.

[Reply] Thank you for your advice. We have made changes according to your advice (line 37-49).

5. Authors wrote, “However, no studies on the prediction of the incidence of JE, which is based on 84 factors such as mosquito density and climate, have been carried out” (pg. 4. line (83-84). In what context the author states this sentence, global or local. Around the globe, researchers have already contributed the stochastic modeling skill for association of JE cases and meteorological parameters (Lin et al., 2012; Pisudde et al., 2017). In what respect the author would like to state that. Justify and clarify.

[Reply] Thank you for your advice. We have made corresponding amendments according to your advice (line 70-79).

6. Line #83-88. Is there only the meteorological factors that affect JE vector density for JE incidences? The several other factors like land use land cover changes, built dam, water bodies, socioeconomic status of the exposed population, intervention schemes/policy. Justify, why authors do not take into account, all these?

[Reply] Thank you for your advice. Due to the problem of data acquisition, our research did not consider these factors that may have a certain impact on the occurrence of JE. We also mentioned these factors in the discussion section, requiring further study and improvement in the future work (line 295-299).

7. Why author opt for stochastic approach over the deterministic approach, the compartment modeling (deterministic) is also another way to model perfectly. Justification needed.

[Reply] Thank you for your advice. We observed that the compartment model was used for some deterministic problems and required idealized hypothetical conditions. Therefore, the existing compartment model has certain defects in predicting the trend of infectious disease (line 75-79).

8. Line #89, instead of Methods, there should be Study Area, Data and Methods. The further subsection may be there.

[Reply] Thank you for your advice. We have made corresponding amendments according to your advice (line 86).

9. The temporal resolution of data is missing in the ‘Data Collection’ section. For the ease of the scientific community, this should be there. A table may be presented here, for data sources of JE incidences as well as meteorological variables, along with the temporal resolution (daily/weekly/fortnightly/monthly/yearly).

[Reply] Thank you for your advice. We have added the corresponding content according to your advice (line 113-117).

10. The models’ outcome in the result section is not expressed much. The author was only direct to see the tables and figures. The crucial observations are not expressed.

[Reply] Thank you for your advice. We have added the corresponding content according to your advice (line 195-204 and line 229-233).

11.The result and methods are very inconsistent. The result outcomes are self-contradictory. The result need to restructure.

[Reply] Thank you for your advice. We have modified the content of the results (line 195-204 and line 229-233).

12. Discussion is poorly written. Author try to make this very lengthy instead of technical discussion and suggestion. The scientific approach is missing in the discussion part.

[Reply] Thank you for your advice. We have made major revisions to the discussion (line 235-299).

13. The conclusion section is missing.

[Reply] Thank you for your advice. We have added the conclusions (line 301-310).

Response to Reviewer #2:

We would like to thank the reviewer for careful and thorough reading of this manuscript and for the thoughtful comments and constructive suggestions, which help to improve the quality of this manuscript.

At the following, the points mentioned by the reviewers will be discussed:

1. The manuscript presents the results of a correlative analysis between selected climate variables and case incidence of Japanese Encephalitis in central China. The paper addresses a topic that is appropriate for the journal and potentially supplies important information for both basic scientific understanding of environmental influences on disease and for potential application to controlling the disease in this area. The paper compares several methods for deriving correlative models between climate and JE incidence, and does in fact provide the reader with an evaluation of the relative effectiveness of these models. However, the paper is frustratingly vague and difficult to follow at times, particularly in its coverage of the basic methodology and in its summary of how the climate variables are related to disease incidence.

[Reply] Thank you for your advice. We have introduced the research methods more clearly (line 121-180), and elaborated on the relationship between meteorological factors, mosquitoes and Japanese encephalitis in the discussion section of the manuscript (line 240-272).

2. The major issue with the paper is that it’s just very difficult to follow exactly what the authors did. Figure 2 shows a flow chart of the methods, showing the sources of data and the general flow of the analysis. It would be helpful to include a table in the body of the paper (rather than the supporting materials) that shows exactly w hat meteorological data are included. There are hints in the paper (temperature extrema seem to be one of the variables) but the reader never really knows for sure, and this becomes a problem later in trying to suss out what the findings of the study really are. All of the data are apparently funneled through the ZIGAM method, but the methods section of the paper never really explains what this is or what it does. It is defined on lines 79-80, and some references are cited. Additionally, in lines 130-141 some additional information is provided. Unfortunately, as a person wholly unfamiliar with this technique, this information was of little use. This may not be the case for readers more familiar with this type of analysis, but for those who are not knowledgeable in this area of analysis, the entire methodology description is not very helpful, at best. Since the entire analysis funnels though this technique, I would strongly urge the authors to write a more complete explanation of what this method does, and why it was chosen for this analysis. Otherwise, the reader is left with the frustrating task of searching through several more papers in order to understand what is in this one.

[Reply] Thank you for careful reading of the manuscript. We have made a table in the body of the manuscript, which can accurately display the included meteorological variables (Table 1, line118). Meanwhile, we have further explained the methods of statistical modeling, including ZIGAM , LSTM and other model (line121-180).

3. I had similar issues with the four methods for developing predictive models, LSTM, BPNN, GBM, and SVR. The way the paper is written seems to suggest the LSTM is somehow considered a standard in this type of analysis, and that the other three methods are presented as potentially superior alternatives? Is this correct? IF so, I would suggest that this be made clearer to the reader. On page 7, the paragraph beginning on line 142, some descriptive material for LSTM is provided, but again, in such a way as to be of little help to an unfamiliar reader. I would also note that there is a lot of detail about the other three methods (BPNN, GBM, and SVR) that is simply glossed over. I have only limited experience with neural networks and gradient boosting, but have worked rather extensively with support vector regression, enough to understand that parameter tuning and kernal basis functions are crucial to understanding the effectiveness of them. Absolutely no indication of any of this information is provided. At minimum, one should report the kernal used and the gamma and r parameters. Ideally, the method for determining these parameters should be provided. I suspect that similar information should be provided about the other two methods (GBM, BPNN). Without this type of information, the analysis cannot be replicated, nor does omitting them help other researcher who may wish to use this approach for similar analyses.

[Reply] Thank you for your advice. We have added the detailed explanation of the parameter setting of SVR, BPNN, GBM and GAM (line 167-180).

4. In addition to the lack of relevant detail in the methods section, the actual results also lack some specificity. The purpose of the paper was to model the relationship between climate and JE incidence, and to predict the occurrence of cases. Judging from Figure 5, the latter goal was achieved. Unfortunately, it is not really clear how it was achieved. Which climate variables were most relevant, and how relevant were they? Hard to really tell from the body of the manuscript. In the abstract (lines 27-30) it is noted that mean temperature and humidity are important (as is density of mosquito species in livestock sheds, a variable whose provenance is not very clear). In the body of the manuscript, though, this information is very difficult to separate out. Clearly, the authors have developed a method that is potentially useful for predicting JE in this study area, and potentially useful for researchers who may want to understand this disease (and possibly other, similar, mosquito-borne diseases) in other places. Without much clearer elucidation of their methods, this would be difficult to do. One of my “acid tests” for reading a manuscript is, “given similar data, could I repeat their methods, even if I had never used them before.” In this case the answer is no. Even if I were to track down all of the literature cited to support the methods, It’s still not clear how the authors reached their conclusions.

[Reply] Thank you for your advice. We have supplemented the content in statistical modelling (lines 121-180). In addition, we have agreed to upload a minimal set of anonymous data. The data of this study can be found at <https://doi.org/10.17026/dans-xm3-7qdm>
