# Supplementary material for: Association between meteorological factors and the prevalence dynamics of Japanese encephalitis
Source: PLoS One. 2021 Mar 3;16(3):e0247980. doi: 10.1371/journal.pone.0247980 (PMC7928514; doi:10.1371/journal.pone.0247980)
Supplement: Attachment — Submitted filename: Response to Reviewers.docx [file pone.0247980.s005.docx]

February 14, 2021

*PLoS ONE*

Dear Editors,

On behalf of my co-authors, we thank you very much for giving us an opportunity to revise our manuscript, we appreciate the editor for their meticulous working attitude.

We have replied the point-by-point to the reviewers' comments and revised the manuscript carefully. the comments of all reviewers are all valuable and very helpful for revising and improving our paper.

We hope that the revised manuscript could meet the high standard of your prestigious journal.

Best regards,

**Taotian Tu** (taotiantu@sina.cn)

Chongqing Municipal Center for Disease Control and Prevention, Chongqing, China;

**Wenge Tang** (twg@cqcdc.org)

Chongqing Municipal Center for Disease Control and Prevention, Chongqing, China.

Response to Reviewer #1:

We would like to thank the reviewer for careful and thorough reading of this manuscript and for the thoughtful comments and constructive suggestions, which help to improve the quality of this manuscript.

At the following, the points mentioned by the reviewers will be discussed:

1. The literature survey still missing the several existing research that have implemented statistical modeling for the association with other Vector Borne Disease like malaria. Author may thoroughly revisit the introduction section and should add some more recent research that focus on Biometeorology. I am suggesting some of the recent findings.

[Reply] Thank you for your advice. We have added most recent publications on Biometeorology in the introduction (line 44, 70, line 345-347 and line 369-371).

2. How do the meteorological variable play role in the life cycle of JE virus Vector, author should must address here.

[Reply] Thank you for your advice. We have added the corresponding content according to your advice (line 252-253, line 276-286).

3. Do different geography have different threshold for the rainfall, temperature, relative humidity, etc. Author should must discuss in the discussion section.

[Reply] Thank you for your advice. We have revised the corresponding content of the discussion (line 289-296).

4. How the build model may be operationalized for public services? Is there any such scope? Brief in short, the application of your model in real time.

[Reply] Thank you for your advice. We have made changes according to your advice (line 299-301, line 330-332).

5. Author say there is a positive effect on the outbreak with a lag 1-month of some meteorological variable. Is this really feasible, as the mosquito complete the life cycle within a month. A justification should be added.

[Reply] Thank you for your advice. We have added the corresponding content according to your advice (line 276-286).
